# Neural cue reactivity and intrinsic functional connectivity in individuals with alcohol use disorder following treatment with topiramate or naltrexone

**DOI:** 10.1007/s00213-025-06745-7

**Published:** 2025-01-24

**Authors:** Warren B. Logge, Paul S. Haber, Tristan Hurzeler, Hugh Gallagher, Henry Kranzler, Kirsten C. Morley

**Affiliations:** 1 Edith Collins Centre for Translational Research in Alcohol, Drugs and Toxicology, Royal Prince Alfred Hospital, Sydney Local Health District, Sydney, NSW Australia; 2 Specialty of Addiction Medicine, Central Clinical School, Faculty of Medicine and Health, University of Sydney, Sydney, NSW Australia; 3 Drug Health Services, Sydney Local Health District, Sydney, NSW Australia; 4 Center for Studies of Addiction, Perelman School of Medicine, Education, and Clinical Center, University of Pennsylvania and Mental Illness Research, Crescenz VAMC, Philadelphia, PA USA; 5 Specialty of Addiction Medicine, Lv 6, King George V Building 83-117 Missenden Rd, Camperdown, NSW 2050 Australia

**Keywords:** Alcohol use disorder, Topiramate, Naltrexone, Functional magnetic resonance imaging, Cue reactivity, Randomized controlled trial

## Abstract

**Rationale:**

Both topiramate and naltrexone have been shown to affect neural alcohol cue reactivity in alcohol use disorder (AUD). However, their comparative effects on alcohol cue reactivity are unknown. Moreover, while naltrexone has been found to normalize hyperactive localized network connectivity implicated in AUD, no studies have examined the effect of topiramate on intrinsic functional connectivity or compared functional connectivity between these two widely used medications.

**Objective:**

This study compared topiramate versus naltrexone on alcohol cue-elicited brain activation and intrinsic functional connectivity in patients with alcohol use disorder.

**Methods:**

Forty-seven participants with alcohol use disorder received daily topiramate (titrating the dose up to 200 mg/day *n* = 21) or naltrexone (50 mg/day, *n* = 26) for at least 6 weeks. Using functional magnetic resonance imaging (fMRI), we examined intrinsic functional connectivity during rest and alcohol cue-elicited neural activation during a visual alcohol cue reactivity task 120 min following treatment administration. Functional connectivity and alcohol cue reactivity and percentage of heavy drinking days (% HDD) associations were assessed.

**Results:**

No differences in either intrinsic functional connectivity or alcohol cue-elicited neural activity were seen between topiramate and naltrexone-treated groups. Overall, participants showed increased alcohol cue-elicited activation in three clusters spanning occipital regions involved in visual recognition of stimuli, and hypoactivation to both alcohol and control cues in three clusters involved in salience attribution and processing of emotional valence of external stimuli. No differences between topiramate versus naltrexone were observed for either functional measure or associations with post-scan % HDD.

**Conclusions:**

Topiramate and naltrexone enacted comparable alcohol cue reactivity and intrinsic functional connectivity patterns. Some overall responses of increased brain activation to alcohol cues in visual processing regions coupled with reduced activation to alcohol and control cues were evidenced for both treatments. These activation patterns were in regions expected to show attenuation of brain activity resulting from treatment. Topiramate and naltrexone may thus enact functional effects through similar modulation of functional neural activity in individuals with AUD.

**Trial registration:**

ClinicalTrials.gov, NCT03479086 https://www.clinicaltrials.gov/study/NCT03479086.

**Supplementary Information:**

The online version contains supplementary material available at 10.1007/s00213-025-06745-7.

## Introduction

Alcohol use disorder (AUD) is a prevalent disorder typically characterised by chronic relapses to heavy alcohol consumption, despite significant clinical impairment or negative consequences (Freyer et al. 2016). Effective treatments including pharmacotherapies exist for problematic drinking and AUD, but the effects of these pharmacotherapies in reducing drinking behaviours are generally modest (Ray et al. 2019) and overall treatment uptake by the community is minimal (Morley et al. 2016). Further development of effective pharmacotherapies is urgently required to provide wider therapeutic options for individuals with AUDs.

Topiramate, an emerging pharmacotherapy for AUD, exerts multiple pharmacologic effects. It enhances GABA-mediated inhibition by modulating GABA_A_ receptors (White et al. 2000) and glutamate through non-competitive antagonism of AMPA/kainate receptors (Ängehagen et al. 2005).Topiramate may thus effectively influence the reinforcing properties of alcohol and associated subjective responses (Miranda Jr et al. 2008). While the specific impact of topiramate on the neurocircuitry of addiction in AUD is not fully understood, it is thought to target established reward pathways, particularly the mesolimbic dopaminergic pathways involving the ventral striatum (Schiffer et al. 2001). Numerous randomized controlled trials (RCTs) have consistently demonstrated topiramate’s efficacy in reducing drinking (Johnson et al. 2003, 2007; Kranzler et al. 2014, 2021) and one meta-analysis involving 1125 patients revealed that topiramate improves drinking outcomes, including heavy drinking reduction and abstinence, with superior effect sizes compared to other alcohol pharmacotherapies such as acamprosate or naltrexone (Blodgett et al. 2014).

Few studies have directly evaluated whether topiramate is superior to naltrexone, one of the widely used and currently approved treatments for AUD. Naltrexone is an opioid receptor antagonist that primarily acts on the µ-opioid receptor and has been shown to reduce the subjective craving of alcohol (Anton et al. 2012; de Wit et al. 1999; Drobes et al. 2004) and heavy drinking outcomes in clinical studies (Jonas et al. 2014; Rösner et al. 2010). There have only been two RCTs comparing naltrexone versus topiramate. One three-arm trial of male alcohol dependent patients compared topiramate with naltrexone and placebo for 12 weeks (Baltieri et al. 2008) and observed a superior effect of topiramate relative to placebo, but no significant benefits over naltrexone. A more recent trial of topiramate versus naltrexone in male and female patients with AUD demonstrated non-significant differences on heavy drinking outcomes but superiority of topiramate on standard drinks per drinking day over time, craving and gamma-glutamyl transferase (Morley et al. 2024).

Functional magnetic resonance imaging (fMRI) cue reactivity paradigms provide a reliable means to assess the impact of pharmacotherapies on diminishing the reinforcing and subjective properties of alcohol and associated cues in AUDs. fMRI studies have consistently revealed that alcohol cues modulate neural activity in mesocorticolimbic pathways and prefrontal networks related to salience, emotion regulation, and reward (Zeng et al. 2021). Changes in brain activation patterns are evident in people with AUD, with neural activity generally increased (Schacht et al. 2013a; Zeng et al. 2021). These functional cue-elicited modulations are associated with behavioural outcomes such as reduced relapse latency (Courtney et al. 2016). Considerably more research has examined the effects of naltrexone on alcohol cue reactivity, demonstrating naltrexone attenuates brain activity in mesocorticolimbic reward-based regions, chiefly the ventral striatum (Mann et al. 2014; Myrick et al. 2008; Ray et al. 2015; Schacht et al. 2013b, 2017), However, few studies have investigated the potential neurobiological mechanisms underlying the efficacy of topiramate in reducing alcohol consumption in AUD. Only one study has examined fMRI neural activation during cue reactivity, using cue videos in twelve topiramate-treated heavy drinkers (up to 200 mg) compared to eight participants taking placebo using arterial spin labelling perfusion fMRI (Wetherill et al. 2021). Scan sessions were completed directly before, and after 6 weeks treatment as part of a larger RCT (Kranzler et al. 2021). Topiramate-treated participants showed reduced alcohol cue-elicited activation in a priori mesolimbic regions-of-interest, including the bilateral medial orbitofrontal cortex (OFC), right OFC, and left ventral striatum. No differences in subjective craving were seen during cue reactivity, but reductions in the medial OFC activation also corresponded with reduced reported craving across treatment. Additionally, reduced activation in medial OFC, right OFC, and right ventral striatum correlated with fewer percentage of heavy drinking days during treatment. Taken together, these suggest topiramate’s attenuation of neural activation within the reward pathways may be a major factor in moderating alcohol consumption in heavy drinkers.

We can also assess functional changes in the brain by examining resting-state (or intrinsic) functional connectivity. This approach examines spontaneous BOLD fluctuations that reflect the inherent neuronal activity in the brain while individuals are awake (Biswal 2012). Intrinsic functional connectivity holds promise as a dependable biomarker for evaluating treatment responses in alcohol and substance use disorders. It can be valuable in the early stages of testing new treatment approaches to gauge their clinical potential (Tolomeo and Yu 2022; Wilcox et al. 2019). To date, no studies have examined changes in intrinsic functional connectivity in AUD associated with topiramate. Some studies have evaluated the effects of naltrexone on intrinsic functional connectivity in AUD (Elton et al. 2019; Morris et al. 2018), but these studies were primarily acute cross-over designs, yielding mixed findings. One study showed naltrexone normalized hyperactive localized network connectivity implicated in AUD (Morris et al. 2018), and another study demonstrated enhanced functional connectivity between areas associated with craving and executive control (Elton et al. 2019) which may explain how naltrexone enacts reductions in consumption in individuals with AUD. However, no intrinsic functional connectivity studies of naltrexone have been conducted in the context of a randomised controlled parallel treatment protocol, which enables evaluation of the chronic effect of a pharmacotherapy enacted at a sustained dose level.

This study aimed to evaluate whether heavy drinkers treated with topiramate versus naltrexone exhibit reduced brain activation to alcohol cues during an fMRI alcohol cue reactivity task and any changes in intrinsic functional connectivity during rest. We hypothesised topiramate would reduce brain activation in areas associated with reward, chiefly mesocorticolimbic regions, compared to naltrexone. This is the first study to investigate the effects of topiramate on intrinsic functional connectivity in AUD. Moreover, only a few studies have evaluated naltrexone using this a functional connectivity approach, and these studies only assessed acute effects. Thus, we applied a data-driven whole-brain parcellation approach to assess evidence of any differences between the two treatments. We also assessed whether cravings for alcohol cues compared to neutral cues differed during cue reactivity task, and whether this was moderated by treatment taken.

## Materials and methods

Participants were randomized to receive topiramate or naltrexone for 12 weeks. The study was conducted over a 48-month period at two sites (Royal Prince Alfred Hospital, North Shore Hospital) in Australia between 2017 and 2022. At approximately week 7 of the trial (*M* = 49 days) participants completed an fMRI cue reactivity task in the MRI scanner. The study was approved by the Human Ethics Review Committee of the Sydney Local Health District (X16–0231 and HREC/16/RPAH/283). The study involved off-label use of a registered medication in Australia and approval was given under the Clinical Trial Notification (CTN) scheme of the Therapeutics Goods Administration (TGA) (2013/0060). All participants included in this MRI sub-study provided written informed consent after randomization for the main trial.

### Participants

Fifty-six participants were recruited as part of a larger trial evaluating the superiority of topiramate treatment compared to naltrexone in alcohol dependence (Morley et al. 2018) with the primary outcomes reported (Morley et al. 2024). Inclusion criteria for that trial included: (i) an AUD diagnosis according to DSM-5 criteria; (ii) Age 18–70; (iii) Adequate cognition and English language skills to give valid consent and complete research interviews; (iv) Willingness to give written informed consent; (v) Abstinence from alcohol for between 3 and 21 days leading up to randomization; (vi) Resolution of any clinically evident alcohol withdrawal (CIWA-Ar; Sullivan et al. 1989). Exclusion criteria were: (i) Active major mental disorder associated with psychosis or significant suicide risk, (ii) Pregnancy or lactation, (iii) Concurrent use of any psychotropic medication other than antidepressants (provided these were taken at stable doses for at least two months); (iv) Currently taking any tricyclic antidepressant; (vi) Use of antiretroviral dolutegravir; (vii) Dependence on any substance other than nicotine; (viii) Clinically decompensated liver disease (jaundice or other signs of liver failure); (ix) History of nephrolithiasis; (x) history of glaucoma; (xi) Lack of stable housing, (xii) Previous hypersensitivity to topiramate or naltrexone; (ix) Any alcohol pharmacotherapy within the past month. For this study, all trial participants were invited to participate with those unfit for MRI scanning excluded and provided specific written consent.

Four participants had a breath alcohol level (BrAC) exceeding 0.00 and their test session was concluded, one participant presented significant cognitive dysfunction, three participants had inadequate neuroimaging acquisition and two participants had significant movement artefacts during cue reactivity and their data not analysed, leaving a sample *N* = 47 participants for cue reactivity (TOP: *n* = 21, NTX: *n* = 26), and *N* = 50 participants for intrinsic functional analyses (TOP: *n* = 21, NTX: *n* = 29). Five participants (TOP: *n* = 1, NTX: *n* = 4) dropped out of the trial without attending their next research appointment after the imaging session. One NTX participant dropped out of the trial, while the remaining four participants (2 for each treatment) missed one or more research appointments and subsequently had incomplete post-scan HDD data, though they completed the trial. These 5 participants were excluded from association analyses with heavy drinking outcomes, with *N* = 44 for cue reactivity and *N* = 45 for resting state functional connectivity remaining with complete 12-week drinking data.

### Procedure

Participants underwent a structured interview and medical consultation on day 0 assessing trial eligibility. For the main trial, participants were randomized 1:1 to a maximal dosage of topiramate of 200 mg/day in two divided doses, with medication titrated gradually upward over 6 weeks from a 25 mg dose once a day at Week 1 to a full dose of 2 capsules (100 mg morning and 100 mg afternoon) from Week 6 until trial end at Week 12. Participants in the naltrexone group took a 25 mg naltrexone dose twice a day (50 mg/day). The half-life of topiramate is approximately 20 h, such that pharmacological steady state is typically reached in five half-lives—or 100 h— amounting to just over 4 days. For this reason, one week after reaching this dose was selected as a suitable time point for these imaging studies when steady state was achieved with confidence. The topiramate dose was selected based on prior evidence of efficacy, tolerability and high completion rates (Baltieri et al. 2008; Miranda et al. Jr 2008) with the dose gradually titrated based on prior evidence this titration approach is well-tolerated (Kranzler et al. 2014).

Naltrexone was administered as a standard dose prescribed for AUD treatment and does not require titration as a competitive opioid receptor antagonist that does not induce the same risks associated with initial dosing seen in many other medications. Naltrexone additionally has a relatively predictable pharmacokinetic profile with a steady absorption and metabolism rate (Avery 2022). Participants then completed baseline questionnaires. Imaging sessions were scheduled at approximately week 7 on the trial and were conducted between 10 am and 3 pm. Participants completed the resting state acquisition approximately 110 min after administration of their first daily relevant medication capsule (100 mg topiramate, naltrexone 50 mg), with the cue reactivity task beginning at 120 min.

### Assessments

#### Baseline (day 0)

Consumption in the preceding 30 days before enrolment was measured using the Timeline followback interview (TLFB) (Sobell and Sobell 1992) in number of Australian standard units (10 g ethanol) per drinking day (henceforth TLFB units). The Alcohol Dependence Scale (ADS) (Skinner and Allen 1982) measured participants’ AUD severity, with higher scores indicating greater severity.

#### Scanning (day 49)

A 7-day TLFB was first completed assessing participants’ previous week of consumption. Immediately prior and after scanning, participants completed an Alcohol Urge Questionnaire (AUQ; Bohn et al. (1995), an eight-item questionnaire measuring current craving and urges to drink, with higher scores indicating greater craving.

### fMRI cue reactivity

A well-established visual cue reactivity task we have used previously (Logge et al. 2021) was used to measure alcohol cue-elicited brain activity. Stimuli comprised three types: 15 alcohol-related pictures depicting types of alcohol and drinking situations (Grüsser et al. 2004; Wrase et al. 2002); a first control type comprising 15 neutral pictures (affective neutral) from the International Affective Picture System (Lang et al. 1997) matched for colour and complexity; and 15 scrambled versions of the alcohol pictures (scramble alcohol), controlling for potential activity related to novelty of neutral images. Images were presented for 6.6 s in blocks of three images of the same type, totalling five blocks per type (alcohol, scramble alcohol, affective neutral). Stimuli and block order were randomized across subjects, and blocks of the same image type did not follow consecutively. Each condition block was preceded by a fixation cross presented for 10 s, modelled as a regressor of no interest. An 11-point visual analogue scale followed each block asking participants to rate their “severity of their craving for alcohol now” from “no craving (0)” to “very severe craving (10)”. Participants selected their craving level on the scale using a Lumina MRI-compatible two-button response pad (Cedrus Corporation; San Pedro, California, U.S.A) within a 10 s window. The cue reactivity task was triggered by MRI scanner pulse to ensure precise temporal equivalence of stimulus presentation and fMRI data acquisition. Participants were debriefed after test session to address potential continued craving elicited during scanning.

### MRI data acquisition

MRI data were acquired on a 3-Tesla GE Discovery (GE Healthcare, Milwaukee, Wisconsin, United States) using a 32-channel head coil. A T1-weighted (1-mm^3 voxel resolution) structural scan was acquired for each subject for screening and registration (TR: 7200 ms, TE 2.7 ms, 176 sagittal slices, 1 mm thick, no gap, 256 × 256 × 256 matrix). For BOLD acquisition, we acquired 203 echoplanar image (EPI) volumes comprising 39 axial slices in an ascending interleaved fashion with a voxel resolution of 1.88 × 1.88 × 2 mm (TR: 3000 ms, TE 30 ms, FA 90 degrees, FOV 240 mm, matrix 128 × 128, acceleration factor 2, slice gap: 1 mm). Participants’ heads were fixated with foam pads to minimize head movement. For fMRI resting-state acquisition participants were instructed to keep their eyes closed and to focus on nothing in particular.

### Image processing

A detailed overview of imaging processing pipeline is provided in the Supplementary Material, with a summary provided here. Anatomical and functional preprocessing was completed using FMRIPrep ((Esteban et al. 2020; Esteban et al. 2019), RRID: SCR_016216), with the two session T1-weighted structural images corrected for intensity non-uniformity, skull-stripped, and then used for reconstruction of the brain surfaces. Volume-based structural images were segmented into white matter, grey matter, and cerebrospinal fluid, and then spatially normalized into MNI space. Functional MRI data for the session per subject and per acquisition (cue reactivity, resting-state) were pre-processed with susceptibility distortion correction using a B0-nonuniformity map (i.e., fieldmap) based on two EPI references in opposing phase-encoding directions, and slice-timing correction was completed. Images underwent motion correction, co-registration to structural data, normalization to MNI space and projection to cortical surface. Functional timeseries were then resampled to FreeSurfer’s (FreeSurfer 6.0.1, surfer.nmr.mgh.harvard.edu) fsaverage space. Cue reactivity data were post-processed using SPM12 (Wellcome Trust Centre for Neuroimaging, London, UK, www.fil.ion.ucl.ac.uk/spm), with functional resampled images smoothed with a Gaussian kernel of 8 mm full-width half maximum (FWHM) to improve sensitivity for group analysis. Resting-state data were further post-processed using eXtensible Connectivity Pipelines (XCP-D) (Ciric et al. 2017; Satterthwaite et al. 2013). Volumes with framewise-displacement (FD) greater than 0.4 were flagged as outliers and excluded. A total of 36 nuisance regressors were regressed out of the BOLD data using the ‘36P’ strategy (Ciric et al. 2017; Satterthwaite et al. 2013). Residual timeseries were then band-pass filtered (0.01–0.08 Hz) and spatially smoothed with a kernel size of 6 mm (FWHM). Fisher’s r-to-z transformation was applied to the Pearson correlation coefficients per cell of the resultant matrices of the ROI-to-ROI connectivity matrix using the Schaefer 17-network 400 parcel atlas (Schaefer et al. 2018) for each subject outputted from the XCP-D processing. Of the 400 parcels from the 17 functional networks, 33 parcels contained missing data and were excluded from second-level analyses (See Supplementary Material for information), leaving a 367×367 symmetrical functional connectivity matrix per participant.

### Statistical analysis

For cue reactivity, imaging data statistical analysis was conducted in SPM12 at two levels. In the first level (subject-specific), two conditions were modelled: alcohol-related (Alcohol) cues; and both control cues combined in a single condition (Control) to control for both novelty and visual complexity of the stimuli, modelled as a box-car function convolved with the canonical haemodynamic response. Motion correction parameters (six regressors) and VAS blocks were also modelled as regressors of no interest within the first-level model. The fixation cross was left as an implicit baseline. The contrast Alcohol > Control was entered into a second-level random-effects whole-brain general linear model (GLM) assessing topiramate and naltrexone group differences in alcohol cue reactivity.

Regions of interest (ROIs) were selected based on areas correlated with alcohol cue reactivity identified by a recent meta-analysis evaluating pharmacotherapy studies in AUD (Zeng et al. 2021) for regions responsive to treatment. Additionally, the left ventral striatum ROI is defined given the region is consistently shown to be reactive to alcohol cues in both topiramate (Wetherill et al. 2021) and naltrexone (Mann et al. 2014; Myrick et al. 2008; Schacht et al. 2013b, 2017) fMRI cue reactivity studies. Nine spherical ROIs (6 mm radius) were used: the left and right caudate, the left and right insula, left and right superior frontal gyrus, right middle frontal gyrus, right precentral gyrus, and left ventral striatum. Further information including ROI MNI locations and visualisations are reported in the Supplementary Material. We used the MarsBar toolbox (Brett et al. 2002) to extract the ROI unweighted beta estimates (*β*) which were then exported into R software (Version 4.0.3) for further analyses using LMMs to assess group treatment differences in ROI activity while accounting for other variables. To optimally balance Type I and Type II error associated with multiple comparisons across ROIs, we accounted for the correlation between the dependent variables of the nine ROIs using the Simple Interactive Statistical Analysis Bonferonni tool (http://www.quantitativeskills.com/sisa/calculations/bonfer.htm). We applied a method outlined in Li et al. (2014), whereby the resultant Bonferroni correction is less stringent as it does not assume the variables are obtained from independent subgroups, as would occur in a standard and more conservative Bonferroni correction. The nine ROI betas had a mean correlation coefficient of *r* =.061 (number of tests *k* = 9), resulting in an equivalent corrected alpha of 0.027 which would be equivalent to a corrected *P* <.05.

All whole-brain analyses not evaluated by the a priori ROIs were conducted using the ‘Multivariate and Repeated Measures for Neuroimaging’ (MRM) toolbox (version 1.0, (McFarquhar et al. 2016) a multivariate and permutation approach which allows advanced statistical modelling of repeated measures mixed effects designs using a multivariate form of the GLM employed within Matlab. First-level betas (Alcohol, Control) were entered into respective second-level full factorial whole-brain general linear models (GLM) comparing topiramate and naltrexone to assess group differences in alcohol and control cue reactivity. Statistical thresholds were set using permutation-based inference, with 5000 permutations conducted to appropriately account for the within and between-subjects’ variance in the model while assessing the betas for the two conditions. Whole-brain analyses were corrected with a family-wise error cluster level inference (pFWEc) used at < 0.05 with a cluster-forming height threshold of *p* =.001, in accordance with recommendations by Eklund et al. (2016).

Resting state data were analysed using CONN toolbox (Whitfield-Gabrieli and Nieto-Castanon 2012) version 22a (Nieto-Castanon and Whitfield-Gabrieli 2021). The 367 × 367 ROI-to-ROI *z* score correlation matrices were entered into a second-level GLM model with between-subjects treatment (topiramate > naltrexone [i.e., 1–1]) evaluating the effect of topiramate versus comparator treatment naltrexone. Age was added as a covariate. A parametric multivariate statistics approach was conducted to target the contributions of the individual ROIs. An FDR-corrected ROI-level p-value (MVPA omnibus test) was used with a threshold of P_FDR_ <0.05 (Benjamini and Hochberg 1995) with a connection (height) threshold of *p* <.01 (uncorrected) to examine the individual connections between the ROIs.

#### Covariates

Number of treatment days prior to scan was added as a control variable as there were variations across patients. Additionally, percentage of heavy drinking days (% HDD) prior to scan day was included as a covariate to control for recent drinking behaviour and to separate associations with prospective drinking post-scan session assessed only within participants with complete HDD data. A major aim of this study was to determine if any alcohol cue-elicited brain activity was related to treatment outcome. We chose % HDD from scan session until trial end as our primary outcome variable rather than time to lapse or time to relapse, which was not considered appropriate given the scanning session was performed seven weeks after trial commencement. HDD is a sensitive, clinically relevant measure in alcohol trials (Falk et al. 2010) defined here as ≥ 4 drinks for females and ≥ 5 drinks for males. While time to relapse is a commonly used endpoint in alcohol use disorder (AUD) trials, it is less suitable for the specific context of this study, given the variability in the timing of participants’ baseline and subsequent scanning days relative to their overall trial participation. This variability, combined with the potential for participants to relapse before the scanning day, renders time to relapse less sensitive and valid for evaluating drinking behaviours across the trial duration. Specifically, time to relapse does not adequately capture the pattern of alcohol consumption following a relapse event or over the course of the trial. By contrast, %HDD provides a continuous measure of drinking intensity and frequency, allowing for a more robust and comprehensive assessment of drinking behaviour across the entire trial period, including post-relapse patterns, and allows the assessment of neural correlates with a continuous drinking outcome, which we have employed in previous studies with other pharmacotherapy targets for AUD (Logge et al. 2021, 2024). A percentage was used here to account for the varying scan day across participants due to scheduling issues from trial start, which was completed approximately during week 7 (mean days = 49). Post-scan % HDD was added as a covariate-of-interest in whole-brain GLMs that included treatment group (topiramate or naltrexone).

Sensitivity analyses were also conducted including covariates for any drinking in the previous 24 h prior to scan (0 = no, 1 = yes), as this can modulate reactivity to alcohol cues and intrinsic connectivity. Current smoking status (0 = no, 1 = yes) was also added as a covariate to these analyses.

## Results

### Sample and drinking characteristics

Table 1 summarizes the demographic and clinical characteristics of the sample and drinking outcomes. There were no significant differences between topiramate and naltrexone for groups for age, gender, years of education, or employment. Both groups demonstrated similar levels craving at baseline represented by the PACS, TLFB drinks per drinking day prior to treatment, number of years since alcohol-related problems began, current smoking, and anti-depressant use (*p*’s > 0.13).

**Table 1:** Sample characteristics with tests of group differences

	Topiramate (*n* = 21)	Naltrexone (*n* = 26)	Test score, (*p*)
*Demographics*			
Age	49.1 ± 11.47	45.96 ± 9.26	*t*=-1.14 (0.26)
Sex, % F	42	26	*W* = 310.5 (0.719)
Education, y	14.9 ± 3.9	14.8 ± 3.4	*t*=-0.09 (0.927)
Unemployed, %	18	17	*W* = 276 (0.892)
*Clinical Characteristics*			
Drinks per drinking day^a^	10.45 ± 6.33	7.29 ± 3.65	*W* = 305 (0.14)
Years since alcohol-related problems began	20 ± 13.42	16.27 ± 10.51	*t =* 0.18, (0.87)
ADS score	18.5 ± 6.81	23.22 ± 7.67	*t =* 1.88, (0.068)
Tobacco use, %	29	50	*W* = 188 (0.066)
Anti-depressant use, %	23	33	*W* = 276 (0.813)
PACS Baseline score	16.21 ± 7.1	18.88 ± 5.01	*t =* 1.39, (0.172)
*Behavioural Characteristics*			
Treatment days before scan day	49.85 ± 12.55	48.32 ± 8.28	*t*=-0.48, (0.638)
Pre-scan % HDD^b^	20.02 ± 31.65	18.05 ± 20.56	*t* = 0.12 (0.908)
Post-scan % HDD^b^	24.9 ± 36.96	21.77 ± 27.89	*t* = 0.08 (0.936)
*Subjective craving*			
AUQ pre-scan	15.2 ± 8.56	15.05 ± 6.86	
AUQ post-scan	16.65 ± 8.78	17.2 ± 6.83	
VAS: Alcohol score	1.84 ± 2.42	1.03 ± 1.56	
VAS: Control score	1.64 ± 1.94	1.18 ± 1.7	

Note Data represent *M* ± *SD*s, unless otherwise noted. ADS = Alcohol Dependence Severity Scale, HDD = Heavy drinking days, AUQ = Alcohol Urge Questionnaire, VAS = Visual Analogue Scale. Two-sample *t-*tests or Wilcoxon Rank Sum tests conducted, where appropriate

^a^ During the 30 days preceding the first day of the study, based on the Timeline Follow-Back method

^b^ Drinking days during the treatment trial

### Subjective craving

Craving was reported both pre- and post-scan with AUQ scores, and during the cue reactivity task using a VAS, with mean scores presented in Table 1. When comparing subjective craving pre and post scan, there were no differences in AUQ craving scores observed pre-scan to post-scan, no treatment differences, or interaction evidenced (p’s > 0.138, see Supplementary Material). For mean scores of VAS ratings following image blocks, the overall sample reported greater subjective craving for alcohol images compared to the control images, F(1,45) = 17.23, *p* <.0001, but there were no treatment differences or interactions seen.

### Alcohol cue reactivity

Apriori ROI analyses assessing alcohol cue reactivity between naltrexone and topiramate found no differences in cue-elicited brain activation for any of the nine ROIs associated with treatment (p’s > 0.057, sensitivity analyses *p* >.061; see Supplementary Material). Whole brain alcohol cue reactivity brain activation regions are reported in Table 2. Across the whole sample, irrespective of treatment group, there was increased neural activation to alcohol cues compared to control cues in large clusters spanning the left and right occipital lobes, and a smaller cluster in the right occipital gyrus, angular gyrus, and precuneus. There was hypoactivation to alcohol and control cues in two clusters, one spanning the left precuneus, mid/posterior cingulate gyrus and parietal lobule, and a bilateral cluster spanning the superior frontal gyrus and anterior cingulate cortex. When assessing brain activation differences between treatment groups, no significant clusters of activation were evidenced for differences in brain activation to alcohol or control cues according to treatment. These effects remained significant in the sensitivity analyses that included further covariates (drinking in the previous 24 h and smoking), except for no longer identifying the cluster in the insula (see Supplementary Material). Overall responses to the alcohol and control cues are also presented in the Supplementary Material.

**Table 2:** Brain regions with BOLD activation changes to fMRI cue reactivity alcohol and control stimuli across participants

Hemisphere	Area	Cluster size	*p*-value (_FWEc_)	X (mm)	Y (mm)	Z (mm)
*Hyperactivation Alcohol > Control*						
L	Occipital Gyrus, Lingual Gyrus, Fusiform Gyrus, Parahippocampal Gyrus, Hippocampus, Thalamus	3921	< 0.001	-44	-80	0
R	Inferior Occipital Gyrus, Fusiform Gyrus,	2460	< 0.001	34	-72	-12
R	Middle Occipital Gyrus, Angular Gyrus, Precuneus	1478	0.003	38	-76	22
*Hypoactivation Alcohol > Control*						
L	Middle Occipital Gyrus, Precuneus, Mid/Posterior Cingulate Gyrus, Parietal Lobule	3820	< 0.001	-30	36	26
L	Anterior Insula, Posterior Orbital Gyrus	229	0.045	-28	14	-14
L/R	Superior Frontal/Superior Frontal Medial Gyrus, Anterior Cingulate Cortex	1331	0.003	-4	56	-2

Note Cluster-corrected at *P*_*FWEc*_ < 0.05

### Intrinsic functional connectivity

We evaluated any significant treatment group differences in intrinsic functional connectivity differences between topiramate and naltrexone. There was no evidence of significant differences in functional connectivity patterns seen at the treatment level, indicating that topiramate did not differ from naltrexone in intrinsic functional connectivity. There were no differences observed when evaluating sensitivity analyses that included previous 24 h drinking and smoking covariates.

### Associations of drinking outcomes and alcohol cue reactivity

Whole brain analyses assessing whether post-scan % HDD was associated with any alcohol cue-elicited neural activation for the cue reactivity task or functional connectivity did not show any evidence of associations with post-scan % HDD drinking outcomes related to the different treatments.

## Discussion

This neuroimaging study compared the effects of topiramate and naltrexone on neural responses during alcohol cue reactivity and intrinsic functional connectivity in patients with AUD. We found that both treatments elicited greater activation to alcohol cues in the primary and secondary visual cortices, consistent with visual processing of sensitized alcohol cues (Zeng et al. 2021). Notably, both naltrexone and topiramate treatments resulted in overall hypoactivation to alcohol and control cues in regions such as the left anterior insula, prefrontal cortex (including the anterior cingulate cortex and superior frontal cortex), and the posterior cingulate cortex/precuneus. These regions are typically associated with increased alcohol-cue elicited activation in AUD (Courtney et al. 2016; Zeng et al. 2021), but show reduced activation after treatment (Holla et al. 2018; Kiefer et al. 2015). The insula, crucial for processing the salience of reward cues (Naqvi et al. 2014), and the anterior cingulate cortex, involved in attentional and memory processes (Jahn et al. 2016), both demonstrated reduced activation post-treatment which has been observed in other treatment studies (Beck et al. 2018; Logge et al. 2021; Myrick et al. 2008). The posterior cingulate cortex/precuneus—important for integrating behaviors and actions (Hayden et al. 2008)—showed similar patterns as observed previously during alcohol cue reactivity (Tapert et al. 2003). However, no significant differences were found between the topiramate and naltrexone groups in brain activation during alcohol cue reactivity. Overall, the observed brain activation patterns align with expected responses to alcohol cues and treatment-related attenuation in these regions.

One study found that 6 weeks of topiramate treatment reduced neural responses to alcohol cues in heavy drinkers with AUD, particularly in the left ventral striatum, bilateral orbitofrontal cortex, and ventromedial prefrontal cortex (Wetherill et al. 2021). Reduced reactivity in the ventral striatum was linked to fewer heavy drinking days (Wetherill et al. 2021). Our study results of generalized cue reactivity correspond with these findings in topiramate, although we did not find any evidence of differences in neural activation to cues compared to naltrexone in the defined mesocorticolimbic ROIs which we hypothesized a priori. A laboratory study examining EEG cue reactivity in smokers with AUD found decreased event-related potentials to both alcohol- and cigarette-related and emotional image cues in high-dose (up to 250 mg/day) topiramate-treated participants group only compared to low-dose (up to 125 mg/day) topiramate or placebo (Robinson et al. 2022). This suggests that topiramate displays a dose-specific generalized dampening of motivational responses to emotional and drug-related cues.

Significantly more research has explored the effects of naltrexone on alcohol cue reactivity, showing that it reduces brain activity in mesocorticolimbic reward regions, particularly the ventral striatum (Mann et al. 2014; Myrick et al. 2008; Ray et al. 2015; Schacht et al. 2013b, 2017), with potential specificity of increased effectiveness in those with high baseline cue reactivity (Bach et al. 2020). Neural response reductions were evidenced in the superior frontal gyri and insula after 1 week in naltrexone-treated alcohol dependent patients (50 mg/day) compared to placebo (Myrick et al. 2008), which correspond with overall alcohol cue reactivity activation patterns in our study findings. Similar results were evidenced in alcohol-dependent participants administered extended-release naltrexone who also showed reduced visual and olfactory alcohol cue reactivity after 4 weeks (Lukas et al. 2013), with attenuated brain activation in cue salience, reward, emotion and learning regions Together, these findings may explain how naltrexone modulates brain function to reduce consumption in AUD. Considering these patterns of neural attenuation seen in naltrexone and topiramate, along with comparable treatment activation and overall cue reactivity, our findings suggest that topiramate and naltrexone similarly attenuate brain activation to alcohol cues. Taken together, neither pharmacotherapy demonstrated a greater effect of further reducing alcohol cue-elicited neural responses after treatment.

This is the first study, to our knowledge, to examine the effects of topiramate on intrinsic functional connectivity patterns in AUD. We found no evidence of treatment differences in intrinsic functional connectivity between topiramate- and naltrexone-treated patients. Acute crossover studies examining effects of naltrexone in AUD have shown a single dose in alcohol-dependent participants normalized a higher local efficiency neural network evidenced during the placebo scan (Morris et al. 2018). Naltrexone also enhanced functional connectivity between regions implicated in craving and executive control in an acute crossover study, with greater effects for drinkers that reported better external locus of control which was attributed to better potential treatment outcomes associated with effective regulation of drinking urges (Elton et al. 2019). No RCTs assessing effects of naltrexone on intrinsic functional connectivity have been conducted in AUD participants, but in treatment-seeking patients with methamphetamine use disorder extended-release naltrexone normalized striatal reward system connectivity (Kohno et al. 2018). The lack of treatment group differences in our current study suggests that topiramate and naltrexone may act similarly to modulate intrinsic functional connectivity. This may involve normalization of highly connected systems that are dysregulated in AUD and substance use disorders. These systems include reward and motivation pathways, salience and attentional networks, and related contributions of other key networks such as the default mode network (see Zhang and Volkow (2019) for a review). This effect may explain overall reductions in alcohol consumption evidenced in the primary trial findings across both treatment groups (Morley et al. 2024). Topiramate showed superiority over naltrexone in some clinical outcomes across the primary trial, including weekly craving, but we did not see a superior effect of either treatment in state-based subjective craving pre/post scan, or any effects during the cue reactivity task here.

This is the first study to include neuroimaging in a pharmacotherapy trial comparing topiramate and naltrexone and including intrinsic functional connectivity. Our study also contained comprehensive subjective craving measures, including state-based craving measured during the cue reactivity task. The similarity in patterns of functional neural activity between naltrexone and topiramate may indicate similar functional effects in the brain at time of scan. Topiramate mechanisms of action in treatment of AUD include normalizing glutamate and GABA-ergic system both directly through potentiation of GABA activity and reducing glutamate receptor excitability, and indirectly affecting the dopaminergic release in the striatum (Guglielmo et al. 2015. Functionally, it attenuates mesocorticolimbic responses to alcohol cues which is also associated with reduced subjective alcohol craving (Wetherill et al. 2021). Naltrexone primarily enacts its effects through opioid receptors, modulating mesolimbic opioidergic activity in individuals with AUD which subsequently reduces the dopamine-mediated rewarding effects of alcohol (Kranzler and Soyka 2018). Taken together, both treatments appear to modulate the mesocorticolimbic pathway implicated in rewarding properties of alcohol, which may explain the similar group effects we have observed across our functional measures. However, another potential explanation is that there are no effects of topiramate and naltrexone on fMRI cue reactivity and intrinsic connectivity in the study. The lack of baseline fMRI cue reactivity reduces the capacity to assess whether there is a reduction in alcohol cue reactivity associated with the treatments. Nevertheless, there is evidence from longitudinal studies of naltrexone that have demonstrated significant reductions in fMRI cue reactivity in those treated with naltrexone, particularly in prefrontal-striatal regions and the inferior frontal gyrus (Bach et al. 2020; Lukas et al. 2013; Mann et al. 2014; Schacht et al. 2013a, 2017)—albeit with heterogeneity in responses and observed more consistently in individuals with AUD that show high cue reactivity at baseline assessment. There is limited evidence in topiramate but reductions in alcohol cue-elicited neural responses have been demonstrated (Wetherill et al. 2021), as well as reductions in other substances such as nicotine (Robinson et al. 2022). Further research employing a longitudinal assessment of treatment responses to topiramate and naltrexone, coupled with the potential biomarker of high cue reactivity at baseline assessment prior to treatment, would be beneficial in demonstrating clear treatment effects for both pharmacotherapies.

The study contained several limitations that limit the generalizability of the findings. As discussed, a significant limitation of this study was the lack of a pre-treatment baseline scan, which reduces our capacity to evaluate change across the treatment trial. A notable limitation of this study is the lack of a formal power analysis, which restricts our ability to determine the probability of detecting true effects given the sample size. While the sample sizes for the two groups are modest, they are comparable to those in other neuroimaging studies investigating treatment trials in alcohol use disorder. Additionally, the neural activation evaluation of a novel target such as topiramate is of significant interest, as it offers insights into the mechanistic actions of pharmacotherapy, even with limited sample sizes (Grodin & Ray, 2019). Future studies with larger cohorts and pre-specified power analyses are warranted to confirm and extend these findings.Additionally, as the trial used naltrexone as the standard of care control to examine the superiority of topiramate in treatment of AUD, there was no placebo treated group. However several RCTs and laboratory studies have investigated the efficacy of topiramate compared to comparator naltrexone for moderate-to-severe AUD (Baltieri et al. 2008; Flórez et al. 2008, 2010) alongside neuroimaging evidence comparing topiramate versus and placebo (Wetherill et al. 2021). Given this existing evidence, and that our study aimed to further elucidate the neural mechanisms underlying its effects—rather than replicate its efficacy relative to placebo—there is sufficient ethical justification (Millum and Grady 2013) for employing naltrexone as a comparator treatment. Another limitation involves the generalizability of the cues employed in the cue reactivity task to an overall appetitive response. The cue reactivity task employed in this study is well-established and has been widely used across several studies evaluating pharmacotherapy responses in AUD (Beck et al. 2018; Logge et al. 2021, 2024), and aligns closely with the cue types implemented in the other neuroimaging cue reactivity study of topiramate (Wetherill et al. 2021)and other key naltrexone fMRI cue reactivity studies (Mann et al. 2014). However, other naltrexone fMRI studies (Lukas et al. 2013; Schacht et al. 2017) have shown the additional benefit of including food and non-alcoholic beverage stimuli to assess the specificity of to alcohol cue responses. Incorporating these stimuli could have provided a more nuanced assessment of alcohol-specific cue reactivity versus general appetitive salience, and may have also revealed how topiramate influences salience associated with food stimuli—particularly considering the reduction in BMI in patients taking topiramate with AUD (Morley et al. 2024) and research demonstrating topiramate is anorexigenic (Moradi et al. 2013; Rosenstock et al. 2007). Evaluating these cue types in future fMRI studies of topiramate would thereby improve the interpretability and generalisability of these findings.

In conclusion, our study demonstrated similar effects of topiramate and naltrexone in modulating neural responses to alcohol cues and intrinsic functional connectivity during treatment for AUD. We observed overall modulations during the fMRI cue reactivity task that indicate both treatments correspondingly attenuated alcohol cue reactivity. This suggests that topiramate and naltrexone may share similar mechanisms in modulating the mesocorticolimbic pathway, which is crucial in responding to the rewarding properties of alcohol. Further research is needed to delineate the distinct and overlapping neural mechanisms of these pharmacotherapies and to explore their long-term effects on neural connectivity and clinical outcomes in AUD treatment.

## Electronic supplementary material

Below is the link to the electronic supplementary material.

Supplementary Material 1

Supplementary Material 2
