# Supplementary material for: Neural cue reactivity and intrinsic functional connectivity in individuals with alcohol use disorder following treatment with topiramate or naltrexone
Source: Psychopharmacology (Berl). 2025 Jan 24;242(7):1641–52. doi: 10.1007/s00213-025-06745-7 (PMC12226611; doi:10.1007/s00213-025-06745-7)
Supplement: Supplementary file 1 — Supplementary Material 1 [file 213_2025_6745_MOESM1_ESM.pdf]

Neural cue reactivity and intrinsic functional connectivity in individuals with alcohol use disorder following treatment with topiramate or naltrexone

Warren B Logge, PhD [1, 2*], Paul S Haber PhD [1,3], Tristan Hurzeler [1,2], Hugh Gallagher[1,2], Henry Kranzler [4], Kirsten C Morley PhD [1,2]

[1] Edith Collins Centre for Translational Research in Alcohol, Drugs and Toxicology, Royal Prince Alfred Hospital, Sydney Local Health District, NSW, Australia

[2] Specialty of Addiction Medicine, Central Clinical School, Faculty of Medicine and Health, University of Sydney, NSW, Australia.

[3] Drug Health Services, Sydney Local Health District, NSW, Australia

[4] Center for Studies of Addiction, Perelman School of Medicine, University of Pennsylvania and Mental Illness Research, Education, and Clinical Center, Crescenz VAMC, Philadelphia, PA, USA.

Supplementary Material

Tables

Table S1: *Supplementary Table 1 Mixed ANCOVA for AUQ scores during fMRI Cue Reactivity*

Table S2:*Supplementary Table 2 Sensitivity Analysis Mixed ANCOVA for AUQ scores during fMRI Cue Reactivity*

Table S3:*Supplementary Table 3 ANCOVAs for ALC > CON contrast across 10 ROIs*

Table S4:*Supplementary Table 4 Sensitivity Analysis ANCOVAs for ALC > CON contrast across 10 ROIs*

Table S5:*Supplementary Table 5 Sensitivity Analysis for Brain Regions with BOLD activation changes to alcohol and control stimuli across participants*

Figures

Figure S1: *Supplementary Figure 1. Visualization of extracted spherical brain Regions of interest (ROIs), displayed on MNI template. Brain slices shown at Z coordinates. ROIs displayed are left (red) and right (dark blue) caudate; left (light green) and right (purple) insula; left superior frontal gyrus, orbital (teal); right superior frontal gyrus (yellow); right middle frontal gyrus (brown), right precentral gyrus (cyan), and left ventral striatum (dark green).*

Methods

Image processing

Anatomical data preprocessing

A total of 1 T1-weighted (T1w) images were found within the input BIDS dataset.The T1-weighted (T1w) image was corrected for intensity non-uniformity (INU) with N4BiasFieldCorrection (Tustison et al., 2010), distributed with ANTs 2.3.3 (Avants et al., 2008) (RRID:SCR_004757), and used as T1w-reference throughout the workflow. The T1w-reference was then skull-stripped with a Nipype implementation of the antsBrainExtraction.sh workflow (from ANTs), using OASIS30ANTs as target template. Brain tissue segmentation of cerebrospinal fluid (CSF), white-matter (WM) and gray-matter (GM) was performed on the brain-extracted T1w using fast (FSL 5.0.9, RRID:SCR_002823, (Zhang et al., 2001)). Brain surfaces were reconstructed using recon-all (FreeSurfer 6.0.1, RRID:SCR_001847, (Dale et al., 1999)), and the brain mask estimated previously was refined with a custom variation of the method to reconcile ANTs-derived and FreeSurfer-derived segmentations of the cortical gray-matter of Mindboggle (RRID:SCR_002438, (Klein et al., 2017)). Volume-based spatial normalization to two standard spaces (MNI152NLin2009cAsym, MNI152NLin6Asym) was performed through nonlinear registration with antsRegistration (ANTs 2.3.3), using brain-extracted versions of both T1w reference and the T1w template. The following templates were selected for spatial normalization: ICBM 152 Nonlinear Asymmetrical template version 2009c ((Fonov et al., 2009), RRID:SCR_008796; TemplateFlow ID: MNI152NLin2009cAsym), FSL's MNI ICBM 152 non-linear 6th Generation Asymmetric Average Brain Stereotaxic Registration Model ((Evans et al., 2012), RRID:SCR_002823; TemplateFlow ID: MNI152NLin6Asym).

Functional data preprocessing

For each of the 2 BOLD runs found per subject (across all tasks and sessions), the following preprocessing was performed. First, a reference volume and its skull-stripped version were generated using a custom methodology of fMRIPrep. A B0-nonuniformity map (or fieldmap) was estimated based on two (or more) echo-planar imaging (EPI) references with opposing phase-encoding directions, with 3dQwarp (Cox and Hyde, 1997) (AFNI 20160207). Based on the estimated susceptibility distortion, a corrected EPI (echo-planar imaging) reference was calculated for a more accurate co-registration with the anatomical reference. The BOLD reference was then co-registered to the T1w reference using bbregister (FreeSurfer) which implements boundary-based registration (Greve and Fischl, 2009). Co-registration was configured with six degrees of freedom. Head-motion parameters with respect to the BOLD reference (transformation matrices, and six corresponding rotation and translation parameters) are estimated before any spatiotemporal filtering using mcflirt (FSL 5.0.9, (Jenkinson et al., 2002)). BOLD runs were slice-time corrected to 1.46s (0.5 of slice acquisition range 0s-2.92s) using 3dTshift from AFNI 20160207 (Cox and Hyde 1997, RRID:SCR_005927). The BOLD time-series were resampled onto the following surfaces (FreeSurfer reconstruction nomenclature): fsaverage. The BOLD time-series (including slice-timing correction when applied) were resampled onto their original, native space by applying a single, composite transform to correct for head-motion and susceptibility distortions. These resampled BOLD time-series will be referred to as preprocessed BOLD in original space, or just preprocessed BOLD. The BOLD time-series were resampled into standard space, generating a preprocessed BOLD run in MNI152NLin2009cAsym space. First, a reference volume and its skull-stripped version were generated using a custom methodology of fMRIPrep. All resamplings can be performed with a single interpolation step by composing all the pertinent transformations (i.e. head-motion transform matrices, susceptibility distortion correction when available, and co-registrations to anatomical and output spaces). Gridded

(volumetric) resamplings were performed using antsApplyTransforms (ANTs), configured with Lanczos interpolation to minimize the smoothing effects of other kernels (Lanczos, 1964). Non-gridded (surface) resamplings were performed using mri_vol2surf (FreeSurfer).

Resting state post-processing of fmriprep outputs

The eXtensible Connectivity Pipeline (XCP) (Ciric et al., 2017; Satterthwaite et al., 2013) was used to post-process the outputs of fMRIPrep version 20.2.7 (Esteban et al., 2020; Esteban et al., 2019), RRID:SCR_016216). XCP was built with Nipype 1.8.5 (Gorgolewski et al. 2011, RRID:SCR_002502). For each of the two BOLD runs found per subject (across all tasks and sessions), the following post-processing was performed. In order to identify high-motion outlier volumes, framewise displacement was calculated using the formula from Power et al. (2014), with a head radius 40.0 mm. Volumes with framewise displacement greater than 0.4 mm were flagged as high-motion outliers for the sake of later censoring (Power et al., 2014). In total, 36 nuisance regressors were selected from the preprocessing confounds, according to the '36P' strategy. These nuisance regressors included six motion parameters, mean global signal, mean white matter signal, mean CSF signal with their temporal derivatives, and the quadratic expansion of six motion parameters, tissues signals and their temporal derivatives (Ciric et al., 2017; Satterthwaite et al., 2013). Finally, linear trend and intercept terms were added to the regressors prior to denoising. The BOLD data were despiked with 3dDespike. Nuisance regressors were regressed from the BOLD data using linear regression, as implemented in nilearn 0.10.0 (Abraham et al., 2014). Any volumes censored earlier in the workflow were then interpolated in the residual time series produced by the regression. The interpolated timeseries were then band-pass filtered using a(n) second-order Butterworth filter, in order to retain signals within the 0.01-0.08 Hz frequency band. The filtered, interpolated time series were then re-censored to remove high-

motion outlier volumes. The denoised BOLD was smoothed using Nilearn with a Gaussian kernel (FWHM=6.0 mm).

Processed functional timeseries were extracted from the residual BOLD signal with Nilearn's (version 0.10.0, (Abraham et al., 2014)) NiftiLabelsMasker was used for the Schaefer 17-network 400 parcel atlas (Schaefer et al., 2018). Corresponding pair-wise functional connectivity between all regions was computed for each atlas, which was operationalized as the Pearson's correlation of each parcel's unsmoothed timeseries. In cases of partial coverage, uncovered voxels (values of all zeros or NaNs) were either ignored, when the parcel had >50.0% coverage, or were set to zero, when the parcel had <50.0% coverage.

Many internal operations of XCP use AFNI (Cox, 1996; Cox and Hyde, 1997), ANTS (Avants et al., 2009), TemplateFlow version 0.8.1 (Ciric et al., 2022), matplotlib version 3.4.3 (Hunter, 2007), Nibabel version 5.0.1 (Brett et al. 2022), Nilearn version 0.10.0 (Abraham et al., 2014), numpy version 1.22.4 (Harris et al., 2020), pybids version 0.15.5 (Yarkoni et al., 2019), and scipy version 1.9.1(Virtanen et al., 2020). For more details, see the xcp_d website https://xcp-d.readthedocs.io.

Regions of Interest

Regions of interest (ROIs) for assessing cue reactivity brain activation were selected based on areas correlated with alcohol cue reactivity identified by a recent meta-analysis evaluating cue reactivity and pharmacotherapy studies in AUD (Zeng et al., 2021). The ROIs used in this study comprise key regions in drug cue reactivity associated with motivational drives and regulation of motivation and salience of drug cues considered to be most likely to show modulations after treatment in responsivity to alcohol cues, while reducing multiple comparisons. Additionally, a left ventral striatum ROI was defined given the region is consistently shown to be reactive to alcohol cues in both topiramate (Wetherill et al., 2021)

and naltrexone (Mann et al., 2014; Myrick et al., 2008; Schacht et al., 2013; Schacht et al., 2017) fMRI cue reactivity studies. The right striatum defined in the naltrexone studies was not implemented due to the similar ventral location to the right caudate ROI identified in Zeng et al's (2021) meta-analysis. Nine spherical ROIs (6 mm radius) were subsequently used: the left and right caudate, the left and right insula, left and right superior frontal gyrus, right middle frontal gyrus, right precentral gyrus, and left ventral striatum. The ROIs are visualized in Supplementary Figure 1 showing the location of the nine ROIs, and the volume of the spherical ROIs were 984 mm^3.

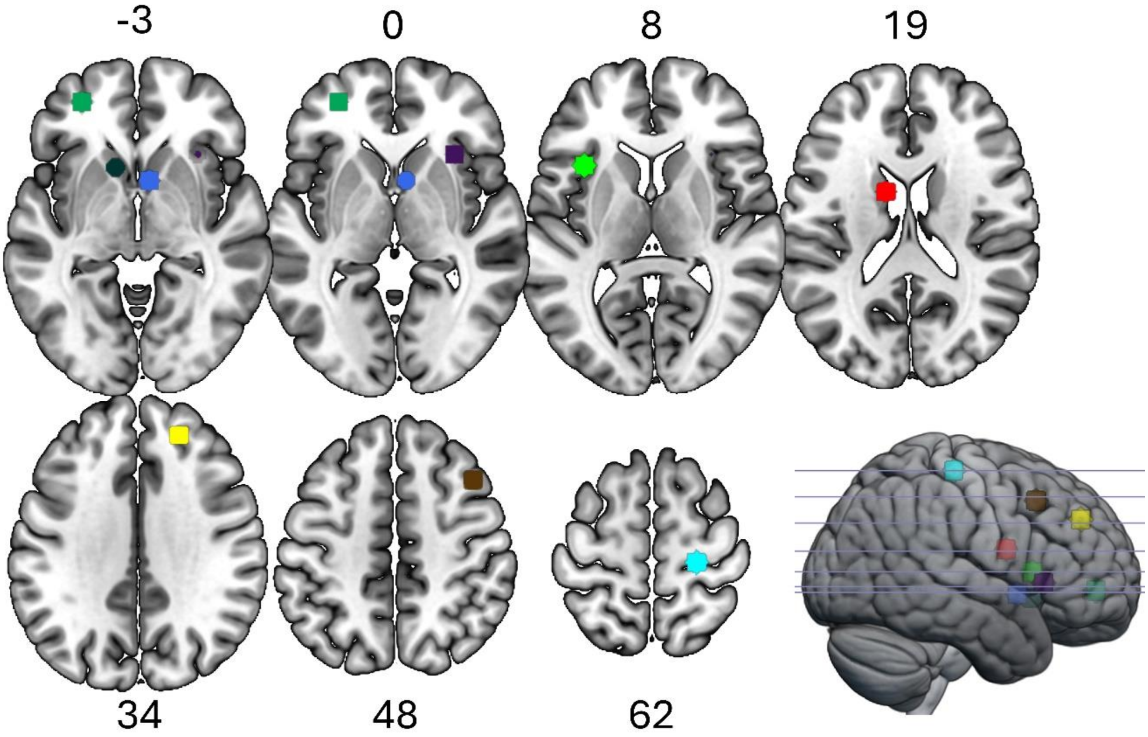

Supplementary Figure 1. Visualization of extracted spherical brain Regions of interest (ROIs), displayed on MNI template. Brain slices shown at Z coordinates. ROIs displayed are left (red) and right (dark blue) caudate; left (light green) and right (purple) insula; left superior frontal gyrus, orbital (teal); right superior frontal gyrus (yellow); right middle frontal gyrus (brown), right precentral gyrus (cyan), and left ventral striatum (dark green).

Supplementary Results

Supplementary Table 1 Mixed ANCOVA for AUQ scores during fMRI Cue Reactivity

Predictors	F(1,42)	p
Age	2.68	.11
Pre/Post Scan	1.3	.261
Treatment	.595	.445
Age * Pre/Post Scan	0.17	.681
Treatment (NAC) * Pre/Post Scan	0.01	.952

Supplementary Table 2 Sensitivity Analysis Mixed ANCOVA for AUQ scores during fMRI Cue Reactivity

Predictors	F(1,42)	p
Age	2.46	.126
Current smoker	0.06	.799
Drinking <24 hr before scan	1.2	.281
Pre/Post Scan	1.24	.273
Treatment	0.205	.654
Age * Pre/Post Scan	0.17	.677
Treatment (NAC) * Pre/Post Scan	0.12	.311
Current smoker * Pre/Post-scan	0.02	.897
Drinking <24 hr before scan * Pre/Post-scan	0.2	.66
Treatment * Pre/Post Scan	0.01	.98

Alcohol Cue Reactivity

Supplementary Table 3 ANCOVAs for ALC > CON contrast across 10 ROIs

Predictors	L Caudate	R Caudate	L Insula	R Insula	L Sup Frontal G	R Sup Frontal G
Treatment	F=0.22, p=.642	F=0.45, p=.507	F=0.01, p=.984	F=1.11, p=.299	F=1.29, p=.262	F=1.53, p=.222
Age	F=3.3, p=.076	F=0.28, p=.6	F=0.87, p=.356	F=0.4, p=.532	F=2.21, p=.144	F=1.14, p=.292

Supplementary Table 3 cont.

Predictors	R Middle Frontal G	R Precentral G	L Ventral Striatum
Treatment	F=3.82, p=.057	F=0.01, p=.934	F=1.01, p=.321
Age	F=0.69, p=.411	F=0.18, p=.673	F=0.81, p=.372

Note. *F*-tests df = 1,42. L = left hemisphere, R = right hemisphere, Sup = Superior, G = Gyrus.

Supplementary Table 4 Sensitivity Analysis ANCOVAs for ALC > CON contrast across 10 ROIs

Predictors	L Caudate	R Caudate	L Insula	R Insula	L Sup Frontal G	R Sup Frontal G
Treatment	F=0.21, p=.648	F=0.45, p=.508	F=0, p=.985	F=1.07, p=.307	F=1.31, p=.259	F=1.47, p=.233
Drinking (<24hr)	F=0.18, p=.676	F=1.59, p=.214	F=0.19, p=.669	F=0.34, p=.563	F=0.9, p=.349	F=0.09, p=.769
Smoking (Yes)	F=0.23, p=.638	F=0.33, p=.567	F=0.07, p=.788	F=0.28, p=.601	F=2.02, p=.163	F=0, p=.949
Age	F=3.24, p=.079	F=0.26, p=.616	F=0.85, p=.361	F=0.37, p=.544	F=2.13, p=.151	F=1.1, p=.301

Supplementary Table 4 cont.

Predictors	R Middle Frontal G	R Precentral G	L Ventral Striatum
Treatment	F=3.7, p=.061	F=0.01, p=.933	F=0.99, p=.326
Drinking (<24hr)	F=0.57, p=.453	F=2.94, p=.094	F=0.64, p=.427
Smoking (Yes)	F=0.06, p=.802	F=0.11, p=.747	F=0.57, p=.453
Age	F=0.67, p=.418	F=0.18, p=.673	F=0.76, p=.39

Note. *F*-tests df = 1,42. L = left hemisphere, R = right hemisphere, Sup = Superior, G = Gyrus.

Exploratory Whole Brain Analyses

Supplementary Table 5 Sensitivity Analysis for Brain Regions with BOLD activation changes to alcohol and control stimuli across participants

Side	Area	Cluster size	p-value ($_{FWEc}$)	X (mm)	Y (mm)	Z (mm)
Hyperactivation Alcohol > Control						
L	Occipital Gyrus, Lingual Gyrus, Fusiform Gyrus, Parahippocampal Gyrus, Hippocampus, Thalamus	3856	.010	-44	-80	0
R	Inferior Occipital Gyrus, Fusiform Gyrus,	2419	.010	34	-72	-12
R	Middle Occipital Gyrus, Angular Gyrus, Precuneus	1464	.010	38	-76	22
Hypoactivation Alcohol > Control						
L	Middle Occipital Gyrus, Precuneus, Mid/Posterior Cingulate Gyrus, Parietal Lobule	3877	.010	-30	84	26
L/R	Superior Frontal/Superior Frontal Medial Gyrus, Anterior Cingulate Cortex,	1202	.010	-4	56	-2

Note. Cluster-corrected at P_{FWEc} < .05.

References

Abraham, A, Pedregosa, F, Eickenberg, M *et al.* (2014) Machine learning for neuroimaging with scikit-learn. *Front Neuroinform* **8**.

Avants, BB, Epstein, CL, Grossman, M, Gee, JC (2008) Symmetric diffeomorphic image registration with cross-correlation: Evaluating automated labeling of elderly and neurodegenerative brain. *Med Image Anal* **12**: 26-41.

Avants, BB, Tustison, N, Song, G (2009) Advanced normalization tools (ANTS). *Insight j* **2**: 1-35.

Ciric, R, Thomas, AW, Esteban, O, Poldrack, RA (2022) Differentiable programming for functional connectomics. *arXiv preprint arXiv:220600649*.

Ciric, R, Wolf, DH, Power, JD *et al.* (2017) Benchmarking of participant-level confound regression strategies for the control of motion artifact in studies of functional connectivity. *Neuroimage* **154**: 174-87.

Cox, RW (1996) AFNI: Software for Analysis and Visualization of Functional Magnetic Resonance Neuroimages. *Comput Biomed Res* **29**: 162-73.

Cox, RW and Hyde, JS (1997) Software tools for analysis and visualization of fMRI data. *NMR Biomed* **10**: 171-78.

Dale, AM, Fischl, B, Sereno, MI (1999) Cortical Surface-Based Analysis: I. Segmentation and Surface Reconstruction. *Neuroimage* **9**: 179-94.

Esteban, O, Ciric, R, Finc, K *et al.* (2020) Analysis of task-based functional MRI data preprocessed with fMRIPrep. *Nat Protoc* **15**: 2186-202.

Esteban, O, Markiewicz, CJ, Blair, RW *et al.* (2019) fMRIPrep: a robust preprocessing pipeline for functional MRI. *Nature Methods* **16**: 111-16.

Evans, AC, Janke, AL, Collins, DL, Baillet, S (2012) Brain templates and atlases. *Neuroimage* **62**: 911-22.

Fonov, VS, Evans, AC, McKinstry, RC, Almli, CR, Collins, DL (2009) Unbiased nonlinear average age-appropriate brain templates from birth to adulthood. *Neuroimage* **47**: S102.

Greve, DN and Fischl, B (2009) Accurate and robust brain image alignment using boundary-based registration. *Neuroimage* **48**: 63-72.

Harris, CR, Millman, KJ, van der Walt, SJ *et al.* (2020) Array programming with NumPy. *Nature* **585**: 357-62.

Hunter, JD (2007) Matplotlib: A 2D Graphics Environment. *Computing in Science & Engineering* **9**: 90-95.

Jenkinson, M, Bannister, P, Brady, M, Smith, S (2002) Improved Optimization for the Robust and Accurate Linear Registration and Motion Correction of Brain Images. *Neuroimage* **17**: 825-41.

Klein, A, Ghosh, SS, Bao, FS *et al.* (2017) Mindboggling morphometry of human brains. *PLoS Comput Biol* **13**: e1005350.

Lanczos, C (1964) Evaluation of Noisy Data. *Journal of the Society for Industrial and Applied Mathematics Series B Numerical Analysis* **1**: 76-85.

Mann, K, Vollstädt-Klein, S, Reinhard, I *et al.* (2014) Predicting Naltrexone Response in Alcohol-Dependent Patients: The Contribution of Functional Magnetic Resonance Imaging. *Alcoholism: Clinical and Experimental Research* **38**: 2754-62.

Myrick, H, Anton, RF, Li, X, Henderson, S, Randall, PK, Voronin, K (2008) Effect of Naltrexone and Ondansetron on Alcohol Cue–Induced Activation of the Ventral Striatum in Alcohol-Dependent People. *Archives of General Psychiatry* **65**: 466-75.

Power, JD, Mitra, A, Laumann, TO, Snyder, AZ, Schlaggar, BL, Petersen, SE (2014) Methods to detect, characterize, and remove motion artifact in resting state fMRI. *Neuroimage* **84**: 320-41.

Satterthwaite, TD, Elliott, MA, Gerraty, RT *et al.* (2013) An improved framework for confound regression and filtering for control of motion artifact in the preprocessing of resting-state functional connectivity data. *Neuroimage* **64**: 240-56.

Schacht, JP, Anton, RF, Voronin, KE *et al.* (2013) Interacting Effects of Naltrexone and OPRM1 and DAT1 Variation on the Neural Response to Alcohol Cues. *Neuropsychopharmacology* **38**: 414-22.

Schacht, JP, Randall, PK, Latham, PK *et al.* (2017) Predictors of Naltrexone Response in a Randomized Trial: Reward-Related Brain Activation, OPRM1 Genotype, and Smoking Status. *Neuropsychopharmacology* **42**: 2640-53.

Schaefer, A, Kong, R, Gordon, EM *et al.* (2018) Local-Global Parcellation of the Human Cerebral Cortex from Intrinsic Functional Connectivity MRI. *Cerebral cortex (New York, NY : 1991)* **28**: 3095-114.

Tustison, NJ, Avants, BB, Cook, PA *et al.* (2010) N4ITK: Improved N3 Bias Correction. *IEEE Trans Med Imaging* **29**: 1310-20.

Virtanen, P and Gommers, R and Oliphant, TE *et al.* (2020) SciPy 1.0: fundamental algorithms for scientific computing in Python. *Nature Methods* **17**: 261-72.

Wetherill, RR, Spilka, N, Jagannathan, K *et al.* (2021) Effects of topiramate on neural responses to alcohol cues in treatment-seeking individuals with alcohol use disorder: preliminary findings from a randomized, placebo-controlled trial. *Neuropsychopharmacology* **46**: 1414-20.

Yarkoni, T, Markiewicz, CJ, de la Vega, A *et al.* (2019) PyBIDS: Python tools for BIDS datasets. *J Open Source Softw* **4**.

Zeng, J, Yu, S, Cao, H, Su, Y, Dong, Z, Yang, X (2021) Neurobiological correlates of cue-reactivity in alcohol-use disorders: A voxel-wise meta-analysis of fMRI studies. *Neuroscience & Biobehavioral Reviews* **128**: 294-310.

Zhang, Y, Brady, M, Smith, S (2001) Segmentation of brain MR images through a hidden Markov random field model and the expectation-maximization algorithm. *IEEE Trans Med Imaging* **20**: 45-57.
